# Adrenal Tuberculosis Presenting as Adrenal Crisis: A Rare Presentation of Common Disease

**DOI:** 10.1155/crie/9086526

**Published:** 2026-06-28

**Authors:** Berhanu Kelemework Fenta, Akberet Semere Yihdego, Bereket Kusse Kurtaile, Worku Ketema

**Affiliations:** ^1^ Departments of Internal Medicine, Yanet Internal Medicine Specialized Center, Hawassa, Sidama, Ethiopia; ^2^ Departments of Internal Medicine, Hawassa University College of Medicine and Health Sciences, Hawassa, Sidama, Ethiopia, hu.edu.et; ^3^ Departments of Pediatrics and Child Health, Hawassa University College of Medicine and Health Sciences, Hawassa, Sidama, Ethiopia, hu.edu.et

**Keywords:** adrenal crisis, primarily adrenal insufficiency, tuberculosis

## Abstract

We report a case of a 45‐year‐old man presenting with hypotension, hyponatremia, hyperkalemia, hypoglycemia and diffuse hyperpigmentation. Monitoring serum cortisol was 0.5 μg/dL, and the computerized tomography (CT) scan showed bilateral adrenal enlargement, consistent with primarily adrenal insufficiency (AI) likely due to adrenal tuberculosis (TB). There was no active pulmonary TB. This case underscores the diagnostic delay and management challenges of TB‐related AI in resource‐limited settings. Even today, clinicians should remain aware of AI due to TB in resource‐limited settings.

## 1. Introduction

Primary adrenal insufficiency (AI) is a rare endocrine disease. It is characterized by a primary adrenal cortical insufficiency, which is distinct from other forms of AI resulting from defects anywhere in the hypothalamic–pituitary adrenal axis [1]. It results from bilateral adrenal cortex destruction leading to decreased production of adrenocortical hormones, including cortisol, aldosterone, and androgens, though the decrement in production can vary as per extent or type of the disease. The incidence is 4 per 1,000,000 population per year in Western countries. It affects women more than men; the ages between 30 and 50 years are commonly affected [2, 3]. While autoimmune adrenalitis is the most common cause in developed countries, tuberculosis (TB) is still the most prevalent etiology in developing countries.

TB is a significant public health concern in Ethiopia, which is one of the nations with a high TB burden. According to WHO estimates, the number of TB cases rose to 156,000 in 2022, and an estimated 21,000 people died from TB [4].

AI usually manifests as an insidious and gradual onset of nonspecific symptoms, often resulting in a delayed diagnosis. The most precise sign is hyperpigmentation of the skin and mucosal surfaces associated with fatigue and weight loss. Though hyperpigmentation is a common presentation (featured in about 70% of the cases) and is a tell‐tale sign, early identification can be challenging in dark‐skinned individuals, which can lead to delayed diagnosis.

In many cases, the diagnosis is made only after the patient presents acutely with vascular collapse, a condition known as adrenal crisis. Adrenal crisis is a potentially deadly condition caused by insufficient cortisol and aldosterone. It is often misdiagnosed and, if inadequately treated, can lead to significant morbidity and mortality [5, 6].

The diagnosis of AI is made by demonstrating low basal and/or stimulated serum cortisol and should be followed by appropriate investigations to establish the underlying etiology. Treatment involves replacement of both glucocorticoids and mineralocorticoids and treating the underlying cause. Another challenge in TB adrenalitis is drug–drug interactions. Rifampicin and adrenocorticoid medications interact, making it difficult to treat both the infection and adrenal cortical insufficiency. Most AI patients caused by TB do not reestablish adrenal function. Patient education is a key feature of the management of this condition. The patient was educated about the need to double the dose of prednisolone in times of stress, infection, and illness, if any [3, 5–9].

This case involves a 45‐year‐old male diagnosed with primarily AI presenting with an adrenal crisis, likely due to adrenal TB, despite the absence of active pulmonary TB.

## 2. An Ethical Review

The patient provided informed consent for the publication of this case report.

## 3. Case Presentation

A 45‐year‐old man came to our clinic with complaints of abdominal pain, nausea and vomiting, postural dizziness, and worsening fatigue of 2 weeks duration. He was being managed for gastritis with Omeprazole 20 mg PO BID for 2 weeks at a nearby healthy facility, but did not show improvement. He had a history of progressive skin darkening, anorexia, and generalized fatigue for 8 months prior to the current presentation. The skin darkening involved the face, oral mucosa, and arms bilaterally, and there was no aggravating or relieving factor. He also lost around 17 kg over an 8‐month period. No history of fever, night sweats, headache, visual loss, diplopia, loss of consciousness, or seizure. There was no history of brain tumor or brain irradiation. No history of exposure to bats was reported. Furthermore, he had no past history of TB, diabetes mellitus, thyroid disorders, or any other chronic medical illness. He is a father of five children, and there is no family history of a similar illness.

**Figure 1:**
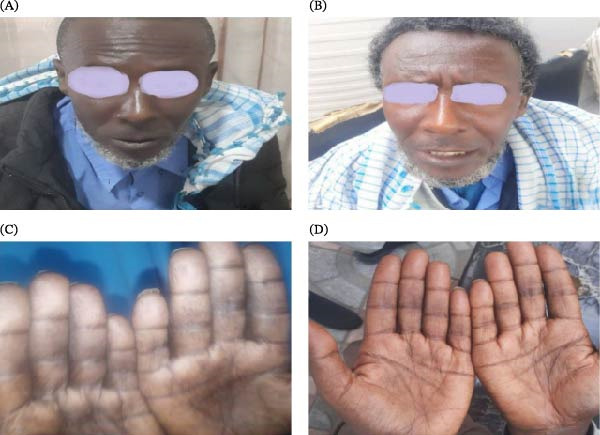
Hyperpigmentation on the hands and face of the patient before (A and C) and after treatment (B and D).

On general physical examination, the patient was conscious and dehydrated. The pulse rate was 120 beats per minute, which was low volume, blood pressure was 112/60 mmHg in the supine position and 84/50 mmHg in the standing position, and a respiratory rate was 22 breaths per min. Generalized hyperpigmentation mainly on the face, palmar creases, elbows, oral mucosa, and anterior leg was noted (Figure 1). The remaining systemic examinations were unremarkable.

## 4. Investigations and Diagnosis

Laboratory investigations showed hemoglobin of 14.1 g/dL, a total leukocyte count of 8.8 × 103/µL (neutrophil of 45%, lymphocytes constitute 48%), whereas the rests are mixed, and a platelet count of 250,000/µL, all of which were normal. The patient was hyponatremic with a serum sodium of 126 mEq/L (reference range, 130–142 mEq/L) and hyperkalemic with a serum potassium of 6.4 mEq/L (reference range, 3.5–5.5 mEq/L). Renal function tests were also deranged with a serum creatinine of 1.54 mg/dL (reference range, 0.55–1.30 mg/dL) and blood urea nitrogen of 40 mg/dL (reference range, 7–18 mg/dL). Random blood sugar was 67 mg/dL at presentation, which is hypoglycemic (reference range, 75–120 mg/dL). The erythrocyte sedimentation rate (ESR) was raised to 70 mm/h (reference range, 0–20 mm/h). Liver function tests were in the normal range. Urine analysis was normal. Echocardiography and Electrocardiography were also unremarkable.

**Figure 2:**
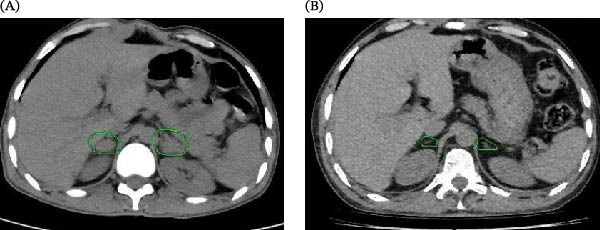
Abdominal computerized tomography scan showing bilateral adrenal enlargement before treatment (A) and reduction in size of adrenal glands after 6 months of treatment (B).

Hence, diagnosis of AI/adrenal crisis was considered based on clinical and biochemical findings described above. Serum cortisol determined at 9:00 am was 0.5 µg/dL, which is lower than the normal ranges and suggests AI. Plasma adrenocorticotropic hormone (ACTH) and cosyntropin test could not be done due to limited resources. Contrast‐enhanced computerized tomography (CT) scan of the abdomen showed bilaterally enlarged adrenal glands with associated surrounding peripheral fat stranding and multiple small enhancing mesenteric nodes (Figure 2). Further diagnostic workup includes X‐ray of the chest, which showed no abnormality. The human immunodeficiency virus (HIV) test was also negative. Initial serum thyrotropin (TSH) level was elevated (11.8 µu/mL), (reference range: 0.3–4.5 mu/L), and became normalized (3.8 mu/L) on follow‐up. The serum free thyroxine (T4) level was normal. Considering the epidemiology as well as laboratory and imaging findings, a working diagnosis of primary AI with an adrenal crisis due to presumably TB adrenalitis was made.

## 5. Treatment and Outcome

The patient was initially managed by dextrose infusion followed by resuscitation with 2 L of intravenous saline infusion over 2 h. Based on different recommendations, this patient was given a stat dose of IV injection of 100 mg of hydrocortisone after completing saline infusion,and then followed by 50 mg of IV hydrocortisone every 6 h for 24 h. The patient showed improvement immediately in terms of fatigue and postural dizziness. The patient was advised on liberal salt intake and discharged with the first line adult dose anti‐TB drugs (daily four tablets of Isoniazid 75 mg, Rifampicin 150 mg, Pyrazinamide 400 mg, and Ethambutol 275 mg for 2 months followed by daily four tablets of Isoniazid 75 mg and Rifampicin 150 mg) for the next 4 months (a total duration of 6 months) and steroid (prednisolone 5 mg PO BID). Worth mentioning, for glucocorticoid replacement, we used prednisolone, and since fludrocortisone is not available for mineralocorticoid replacement, the patient was advised on liberal salt intake and a high‐potassium‐containing diet restriction. The patient was also educated about the need to double the dose of prednisolone in times of stress, infection, and illness, if any.

During subsequent follow‐ups, serum sodium, potassium, and TSH were within normal limits. After 6 months of therapy, the patient also gained around 11 kg, and skin hyperpigmentation started to improve (Figure 1). CT scan repeated after 6 months of therapy showed decreased lymphadenopathy, and also there was a reduction in the size of both adrenal glands (Figure 2). The dose of prednisolone was reduced after anti‐TB completion, and the patient is currently taking prednisolone 5 mg morning and 2.5 mg in the evening. The patient did not complain any adverse drug reaction thus far.

## 6. Discussion

Primary AI is a relatively rare endocrine disorder whose morbidity and mortality largely result from delayed diagnosis or inadequate steroid replacement; it may arise from autoimmune causes, infections (notably TB and HIV), adrenal tumors, or vascular and infiltrative diseases. Historically, TB was the leading cause worldwide and remains the most common cause in developing countries, whereas autoimmune adrenalitis (Addison’s disease) is the predominant cause in the Western world—60%–70% of which is associated with autoimmune polyglandular syndrome (APS). Importantly, TB must still be considered in developing settings because treating active TB with steroids alone can have grave consequences. TB can cause AI in diverse mechanisms: primary AI due to direct involvement of the adrenal gland, secondary AI due to prolonged steroid therapy in some forms of TB, and subclinical steroid deficiency unmasked by anti‐TB therapy‐related enzyme induction. The primary AI due to TB adrenalitis manifests when more than 90% of the bilateral adrenal glands have been destroyed by tuberculous lesions; hence, adrenal function usually does not fully recover after treatment. Like in our case, ~25% of the patients with TB adrenalitis have no other organ involvement [4, 10, 11].

Because the presenting symptoms of AI are nonspecific, diagnosing the condition can be problematic, particularly in resource‐limited settings. When gastrointestinal problems and weight loss are the primary worries, AI will not be a major part of the differential diagnosis. The possibility of an adrenal crisis makes an early diagnosis of the disease crucial. After a CT scan revealed bilateral adrenal enlargement, we were able to identify AI as the most likely cause of our patient’s symptoms. Before ordering radiological imaging, cortisol levels might have been checked, and AI may have been evaluated sooner, given the patient’s symptoms. In summary, AI due to TB adrenalitis generally manifests as nonspecific symptoms with an insidious beginning. This increases the likelihood of an adrenal crisis as the diagnosis is often missed.

AI often presents with nonspecific symptoms, requiring strong clinical suspicion for diagnosis. Biochemical clues include hyperkalemia from mineralocorticoid deficiency and hyponatremia from sodium loss, volume depletion, and cortisol‐related vasopressin increase. Some patients show elevated TSH, and adrenal disease should be suspected in hypothyroid patients whose symptoms worsen after thyroxine. Subclinical TSH elevation may normalize with glucocorticoid therapy, but persistent elevation warrants evaluation for APS.

A morning serum cortisol level higher than 500 nmol/L (18 µg/dL) usually excludes adrenal disease, while a level below 165 nmol/L (6 µg/dL) is suggestive of AI [7].

However, most patients will need a short synacthen test for confirmation or exclusion of AI. This involves injecting 250 µg of Synacthen (tetracosactrin; synthetic analog of ACTH intramuscularly or intravenously). If the cortisol response to synacthen is inadequate, the plasma ACTH level should be measured. A raised plasma ACTH level confirms the diagnosis of primary adrenal disease, whereas patients with secondary AI due to pituitary or hypothalamic disorders have a low or inappropriately normal plasma ACTH level [5, 7]. We could not practice this because ACTH testing was unavailable at our center. An abdominal CT scan should be performed in all newly diagnosed cases of AI because information obtained by the CT scan is so important in the etiological diagnosis of AI. Patients with active TB or recently acquired disease (<2 years) usually have noncalcified bilateral adrenal enlargement, while calcification and atrophy are the usual findings with more remote infection or inactive disease. Adrenal TB can be diagnosed if there is bilateral adrenal enlargement associated with necrotic areas, with or without calcification, and evidence of TB is also found in other organs (e.g., lungs). Whenever possible, tissue specimens should be obtained for analysis, especially in cases in which the adrenal involvement is the only evidence of TB. Histopathological examination revealed granulomatous inflammation and caseous necrosis. Other causes of AI associated with adrenal enlargement should be differentiated from adrenal TB. These conditions include adrenal metastasis, non‐Hodgkin’s lymphoma, adrenal hemorrhage, histoplasmosis, and primary adrenal tumors [12].

Other causes of primary AI with bilateral adrenal enlargement were epidemiologically unlikely, though histopathological confirmation was required. Opportunistic fungal infection was excluded by a negative HIV status and absence of systemic features. In male patients, congenital adrenal hyperplasia and adrenal leukodystrophy are considered when glands are normal or small; however, bilateral enlargement, recent onset, patient age, and lack of family history rendered these diagnoses improbable [12].

Treatment of adrenal TB includes early initiation of anti‐TB and steroid therapy. For patients who present with an adrenal crisis, appropriate samples for ACTH and cortisol should be taken before corticosteroid therapy is given. Patients with suspected adrenal crisis should be treated with an immediate parenteral bolus injection of 100 mg of hydrocortisone, followed by appropriate fluid resuscitation and 200 mg of hydrocortisone/24 h (via continuous IV therapy or 6‐hourly injection) till patient stabilizes [5, 7]. Mineralocorticoid replacement therapy with fludrocortisone 50–100 µg daily can be initiated once the daily hydrocortisone dose has been reduced to <50 mg because at higher doses, hydrocortisone provides sufficient stimulation of mineralocorticoid receptors [5]. Hydrocortisone is the ideal glucocorticoid since its action mimics the endogenous cortisol rhythm. The dose is 15–25 mg PO qDay in two to three divided doses. The higher dose should be taken as soon as the patient is awake in the morning, and the last dose to be taken 4–6 h before bedtime [5, 7].

Concurrent treatment of AI and TB can be challenging as rifampicin is a strong inducer of the cytochrome P450 (CYP) system, involved in the metabolism of adrenocorticoids. This interaction results in lower plasma levels of steroid drugs, which could potentially be dangerous. No exact guidelines for dose adjustment are available, but it is advised that the dose of steroids should be adjusted when the enzyme inducer is started and stopped [11]. In our case, we doubled the dose and informed the patient to even increase the dose if there is an intercurrent infection or stress. When starting anti‐TB treatment, the literature suggests increasing the dosage of steroid drugs three times (hydrocortisone) and twice (fludrocortisone). Nevertheless, since we were unable to obtain these medications, we were forced to use a high dose of prednisolone, which we eventually tapered after the anti‐TB treatment had concluded. We have not noticed any adverse events thus far [5].

Regular monitoring for under‐ and overreplacement is essential. Patients should be reviewed every 3 months and then annually once stable. Mineralocorticoid adequacy is assessed clinically (salt craving, postural hypotension, and edema) and by serum electrolytes and plasma renin. Insufficient fludrocortisone may cause hypotension, hyponatremia, hyperkalemia, poor weight gain, and salt cravings; excess causes the opposite. Patient education on these symptoms is vital. Hypertension on fludrocortisone may warrant dose reduction. Glucocorticoid replacement should be monitored clinically, including weight, postural BP, energy, and signs of excess [5, 7].

Regarding anti‐TB initiation, it is critical to determine whether TB is in the active stage. If TB focuses are in the active stage, regular anti‐TB therapy should be given for 6–18 months. In view of the fact that glucocorticoid replacement therapy can make old TB become active or spread the active TB focuses, anti‐TB therapy should be routinely administrated for about 6 months after the initial diagnosis of adrenal TB [12, 13]. Adrenal enlargements usually resolve with anti‐TB treatment, while AI may not recover in most cases [11]. A retrospective analysis of 25 cases of adrenal disease caused by adrenal TB in Tibet showed that 19% of patients successfully discontinued glucocorticoid replacement therapy within 1 year after regular anti‐TB therapy. Glucocorticoid replacement therapy was successfully discontinued in 4% of patients with a course of 1–4 years and in 4% of patients with a course of more than 4 years [12]. Therefore, early diagnosis and timely institution of anti‐TB treatment can be life‐saving.

Adrenal crisis is a life‐threatening emergency that contributes to excess mortality in AI. Guidelines emphasize urgent initiation of therapy when a crisis is suspected. Diagnostic tests must not delay treatment: blood samples for cortisol and ACTH can be drawn immediately, but therapy should begin without waiting for the results. In acutely ill patients, diagnosis can be confirmed safely after clinical recovery [7, 9, 14].

Barriers to TB adrenalitis diagnosis and management in our setting include nonspecific symptoms, limited access to confirmatory tests (cosyntropin stimulation and serum ACTH), and lack of optimal therapies (oral hydrocortisone and fludrocortisone). The cosyntropin test evaluates adrenal function and HPA axis integrity, with adequacy defined by peak cortisol at 30–60 min [12].

It is noteworthy that, although a definitive diagnosis of TB adrenalitis was not established, several points regarding the management of this case deserve empahsis. Urgent steroid replacement was required to address AI. However, the necessary glucocorticoid dose carried a risk of immunosuppression. Conseuently, antiTB was initiated simultaneously, as TB remained a significant clinical possibility.

This case report has its own strengths and limitations. Notably, this is a presumable adrenal TB diagnosis in a resource‐limited setting with a negative chest X‐ray and sputum findings and subsequent responses to our empiric management. However, the pathological diagnosis was not confirmed by an adrenal gland biopsy, which was the major shortcoming of this case. Serum cortisol level was measured after the patient has taken Hydrocortisone.

## 7. Conclusion

We report a case of primary AI due to presumably adrenal TB. The patient had no active findings in the lung, but he responded well to fluid resuscitation, IV Hydrocortisone, anti‐TB, and prednisolone therapy. Given the current status of TB in Ethiopia, in particular and developing countries in general, particular attention should be directed to its role in causing primary AI. Moreover, delayed diagnosis often leads to increased mortality and morbidity; thus, physicians should keep a high index of suspicion for diagnosis of TB‐induced adrenal crisis.

## Author Contributions

All the four authors made a significant contribution to this case report and hence fulfill the criteria of authorship.

## Funding

The authors have nothing to report.

## Conflicts of Interest

The authors declare no conflicts of interest.

## Data Availability

The data used to support the findings of this report will be available from the corresponding author upon a reasonable inquiry.
